# mrSNP: Software to detect SNP effects on microRNA binding

**DOI:** 10.1186/1471-2105-15-73

**Published:** 2014-03-15

**Authors:** Mehmet Deveci, Ümit V Çatalyürek, Amanda Ewart Toland

**Affiliations:** 1 Biomedical Informatics, Computer Science and Engineering, The Ohio State University, Columbus, Ohio, USA; 2 Biomedical Informatics, Electrical and Computer Engineering, The Ohio State University, Columbus, Ohio, USA; 3 Molecular Virology, Immunology and Medical Genetics, The Ohio State University, Columbus, Ohio, USA

**Keywords:** miRNA, SNP, mRNA, microRNA binding

## Abstract

**Background:**

MicroRNAs (miRNAs) are short (19-23 nucleotides) non-coding RNAs that bind to sites in the 3’untranslated regions (3’UTR) of a targeted messenger RNA (mRNA). Binding leads to degradation of the transcript or blocked translation resulting in decreased expression of the targeted gene. Single nucleotide polymorphisms (SNPs) have been found in 3’UTRs that disrupt normal miRNA binding or introduce new binding sites and some of these have been associated with disease pathogenesis. This raises the importance of detecting miRNA targets and predicting the possible effects of SNPs on binding sites. In the last decade a number of studies have been conducted to predict the location of miRNA binding sites. However, there have been fewer algorithms published to analyze the effects of SNPs on miRNA binding. Moreover, the existing software has some shortcomings including the requirement for significant manual labor when working with huge lists of SNPs and that algorithms work only for SNPs present in databases such as dbSNP. These limitations become problematic as next-generation sequencing is leading to large numbers of novel variants in 3’UTRs.

**Result:**

In order to overcome these issues, we developed a web-server named mrSNP which predicts the impact of a SNP in a 3’UTR on miRNA binding. The proposed tool reduces the manual labor requirements and allows users to input any SNP that has been identified by any SNP-calling program. In testing the performance of mrSNP on SNPs experimentally validated to affect miRNA binding, mrSNP correctly identified 69% (11/16) of the SNPs disrupting binding.

**Conclusions:**

mrSNP is a highly adaptable and performing tool for predicting the effect a 3’UTR SNP will have on miRNA binding. This tool has advantages over existing algorithms because it can assess the effect of novel SNPs on miRNA binding without requiring significant hands on time.

## Background

MicroRNAs (miRNAs) are predicted to regulate over 60% of all genes and as such have a significant impact on cell function and biology [1]. MiRNAs bind to the 3’UTR of an mRNA which results in decreased expression of the targeted gene. Thus, miRNA binding analysis is essential for any biological workflow that examines gene expression.

Processing of miRNAs is a multi-step process. First the miRNA transcript folds into a hairpin loop which is called the pri-miRNA. The hairpin loop is processed further into a pre-miRNA and is exported to the nucleus where it binds with dicer and is processed into a mature miRNA of roughly 19-23 nucleotides in length. The mature miRNA together with the protein-silencing complex (RISC) seeks and binds to mRNA at target sites. Binding can cause mRNA destabilization leading to translational repression or direct degradation of the mRNA target. Initially, miRNA targets were detected through classical genetic techniques. Due to the painstaking nature of these experiments and the lack of high-throughput protocols, there is a great need to develop computational techniques to determine miRNA targets. After it was shown that 3’UTR regions contain binding sites for miRNAs that have some degree of complementarity, various methods of computational predictions were developed.

Generally, plant miRNAs have perfect base-pairing with their target, causing its degradation. In animals, miRNAs can also form limited base-pairing, primarily between the 2nd and 7th bp from the 5’ end of miRNA (seed of miRNA), which leads to translational repression. This imprecise sequence matching makes it more difficult to predict miRNA targets in animals with high accuracy. Different techniques have been proposed to predict mammalian miRNA-mRNA binding. These include the pattern of base pairing, thermodynamic stability of the miRNA-mRNA hybrid, comparative sequence analysis for conservation, and examination of multiple target sites [2].

Several software programs have been developed that utilize one or more of these methods to identify miRNA binding sites in the genome. TargetScan checks thermodynamic stability and conservation of the target sites in related species [3]. Miranda combines the pattern of base pairing, the thermodynamic stability of the miRNA-mRNA hybrid and comparative sequence analysis for conservation [4]. RNAhybrid determines the optimal and subobtimal binding energies between a given miRNA and its mRNA target [5]. MicroInspector detects binding sites according to complementarity using two sliding windows of 6 nucleotides in length [6]. Pictar requires base-pair matches in the seed region of miRNA, applies filtering by calculating thermodynamic binding energy, and assigns a likelihood score using a Hidden Markov Model for each binding [7]. Diana-microT considers principles of binding energy and conservation [8,9]. It also integrates biological pathways and analysis of interactions between predicted target genes.

Disease-associated functional SNPs may alter gene expression. Therefore, the relationship between SNPs and miRNAs becomes important for understanding the role of SNPs on disease [10]. Although there are many miRNA binding prediction tools that have been studied in the last decade, fewer studies to assess SNP effects on miRNA binding have been published [11-15]. Recently, the databases microSNiPer, Patrocles, Mirsnpscore, miRdSNP, MirSNP, PolymiRTS have been released [16-21]. These databases follow similar algorithms as those utilized by the miRNA prediction tools in order to detect the effects of the SNPs on miRNA binding. These algorithms are run on the whole genome for all SNPs present in a genomic database like dbSNP, then results are stored. Users can query the results using SNP, gene or miRNA IDs. One of the deficiencies of these databases is that they only work for SNPs that already exist in databases and do not work for novel or unreported SNPs. Moreover, if the list of SNPs is large, the web interface of the tools may require an infeasible amount of manual labor. With the advent of next-generation sequencing technologies such as RNA-Seq, exome and whole genome sequencing, thousands of novel SNPs in 3’UTRs are being identified. RNA-Seq, which sequences all expressed genes in a sample, provides concordant gene expression and SNP data. Since a substantial number of the detected SNPs are previously undocumented, use of algorithms that require a SNP to be present in dbSNP may not meet the needs of researchers using RNA-Seq or other next-generation sequencing methods. Currently, when a novel SNP is encountered, a user can compare the location of the SNP against the predicted and validated miRNA target sites using the current prediction tools which is fairly labor intensive. The probability of the SNP disturbing a binding site can be considered to be proportional to the distance of the SNP to the seed of the target site. However, a SNP may not affect binding even when it is very close to the miRNA target seed region. Moreover, a SNP may introduce a totally new binding with a new miRNA, which is impossible to capture with the current databases. Thus, next-generation sequencing data demands new computational tools to relate the SNP and gene expression, which motivated us to develop a web-based tool, named mrSNP, to overcome the shortcomings of existing tools.

## Implementation

**Figure 1:**
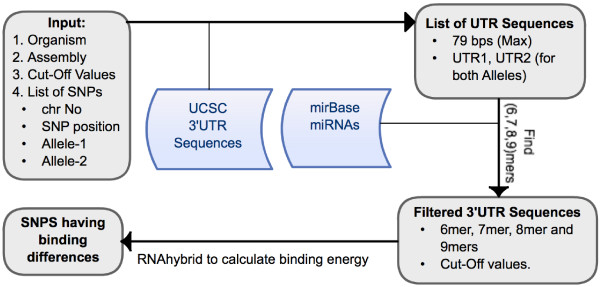
The workflow of mrSNP software.

The implementation of the mrSNP is presented in Figure 1. All the 3’ UTR sequences and phastCons scores of the each nucleotides are downloaded from the UCSC Database using the Genome Table Browser [22]. Each chromosome is stored in a single file, where each sequence has information including gene name and 3’UTR sequence coordinates. All available miRNAs are downloaded from the mirBase database and clustered according to their conservation across species using the information obtained from the TargetScan prediction tool [3,23]. The software accepts input SNPs with the related information containing the organism, the assembly according to which the mapping is done, the chromosome on which the SNP is located, the position of the SNP in the given chromosome, and the SNP alleles. Once this information is provided, the software searches for the sequence where it is located. If SNP is not located in a denoted 3’ UTR sequence, no further calculation is done and the software reports the SNP as, “not in 3’UTR”. If the SNP is found in a 3’UTR, the 79 basepairs (bp) of sequence that contains the SNP at the center is returned at this step. This length (79bp) was chosen based on the observation that the typical maximum size of an miRNA is 25 bp and a maximum 15 bp loop is allowed in the binding. Therefore, we allow a miRNA binding site to have a maximum length of 40 bp. If a SNP is to affect miRNA binding, it will be located in the miRNA’s binding site whose start/end nucleotide can be at most 39 bp apart from the SNP. Therefore, a 79 bp sequence (40 bp + 39 bp) is the minimal sequence to use for calculating potential miRNA binding differences. Once this sequence is obtained, it is duplicated and each SNP allele is substituted in the correct position. After this point, for each miRNA stored we check the existence of a minimum of 6 consecutive Watson-Crick (W-C) matches starting from second position of the miRNA seed region.

The remainder of the approach is adapted from [9]. A sequence with 6 (7, 8, or 9) consecutive matches is called a 6mer (7mer etc.). We allow a single G:U wobble for 7, 8 and 9mer sequences. If no instances satisfy matching criteria, the miRNA and the sequence couple are not investigated further, and we conclude that the miRNA does not bind to this region. Moreover, if the sequence has at least 7 Watson-Crick matches in the seed region, it is considered as a miRNA binding site immediately. For weaker bindings such as the 6mers, or 7, 8 and 9mer sequences containing a single G-U wobble, we calculate the binding energy with RNAhybrid [5]. RNAhybrid runs a dynamic programming algorithm that finds the suboptimal binding energy between 2 sequences. For 6mers and 7mers (8mers and 9mers), we say that microRNA binds to a sequence if its binding energy is higher than 74% (60%) of the maximum binding energy. The numbers and methods used are adapted from [9]. For a given SNP-miRNA couple, the steps explained above are followed for both of the SNP sequences. If one of the them satisfies the binding criteria, while the other does not, we report this as a binding difference.

In the literature, many of the prediction tools apply a post-processing step to reduce the false positive rate of the binding predictions. This is performed using the conservation of the target site across different species. If the target site is conserved over different species, the binding possibility is considered to be higher. Although mrSNP does not filter out the results with this post-processing method, it calculates the conservation score (CS) of the seed region using the phastCons scores provided by UCSC database. For each prediction, CS is obtained as the average phastCons score of the nucleotides in the seed region. Then, it reports the probabilistic CS of the seed region as well as the conservation of the miRNA over the species.

## Usage

mrSNP software is publicly available from http://mrsnp.osu.edu. First, the user selects the organism and the assembly used in the analysis. mrSNP currently supports 11 organisms with the available assemblies.

Once organism and assembly are chosen, the user inputs the list of SNPs by either typing in the textbox or uploading a file. Each line should contain a single SNP with chromosome number, SNP position, and first and second allele. Each entry is separated by a space. An e-mail address can be provided for obtaining results. Also, the cut-off ratios to apply 6, 7, 8 and 9mers are parametrized for the option of using different ratios for different organisms. When a job is submitted, the user is directed to another page summarizing inputs and a link to the results page. Once the result is ready, it is displayed in a table containing the fields: chromosome, SNP position, target gene, the binding miRNA, the binding energy difference, the binding energies of each SNP, the cut-offs applied to each sequence, and the alignment of the bindings. If a SNP is not located in any 3’UTR region, or if it does not affect any miRNA bindings, the related information is reported at target gene field. A downloadable file is also provided. If there are any errors found, a link to the error page is given at the bottom of the page.

## Results and discussion

Although mrSNP does not require its input to be validated SNPs, in order to evaluate the accuracy of mrSNP, we ran a series of experiments on multiple sets of experimentally validated SNP-miRNA couples for human hg19 assembly.

In the first set of validation experiments, we ran experimentally validated disease associated SNPs used in the experiments of [20] in order to compare mrSNP with different databases. 16 SNPs

which are both associated with disease and experimentally validated to disrupt miRNA binding were chosen. Table 1 gives the results of this experiment. For each SNP-miRNA pair, the table reports the SNP’s rsID, the name of the miRNA, the location of the SNP, SNP alleles, the success/failure of mrSNP, and the explanation of the behavior. In this experiment, mrSNP is able to recover 11 disease associated SNPs out of 16. Among the 5 SNPs which are predicted not to affect miRNA bindings, the effect of rs13212041 on *hsa-miR-96* is not captured as the SNP is not located in the 3’UTR. The effects of 3 SNPs (rs2735383, rs34764978, rs9341070) are not recovered as the sequences for both of the alleles do not satisfy the minimum matching criteria. On the other hand, although mrSNP recognizes the binding energy change of *hsa-miR-148a* for rs67384697; both of the sequences satisfy the matching criteria, and no effect on miRNA binding is detected. Among the 11 SNP-miRNA pairs that are successfully detected by mrSNP, 7 of them are captured because the SNPs break a matching in the seed region which causes the sequence not to meet the minimum matching criteria. The other 4 SNPs introduce GU wobbles in the seed region. The binding of these SNPs are predicted to be disrupted since the binding energies are calculated to be lower than the required threshold. In comparing the results of mrSNP to other databases and algorithms described in [20], MirSNP, PolymiRTS, Mirsnpscore, and Patrocles are able to capture 12, 7, 7, and 5 of the disease-associated SNPs respectively. Thus, mrSNP outperforms all tools except MirSNP. MirSNP detected similar binding differences as mrSNP with the exception of capturing the rs67384697 - *hsa-miR-148a-3p* pair. MirSNP reports this pair as “Enhance/Decrease” which means a binding energy difference between two sequences for each the allele of the SNP was measured, rather than a break in the binding. As explained in Table 1, a binding energy difference between the alleles is also captured by mrSNP, however, it is not reported because both of them satisfy the matching criteria.

**Table 1:** **Results of mrSNP on 16 experimentally validated disease-associated SNPs described in [**[20]**]**

**SNP**	**miRNA**	**Chr**	**Position**	**Alleles**		**Success**	**Explanation**
rs1063320	hsa-miR-152	6	29798749	C	G	✓	Binds one of the sequences with 7 consecutive matches. SNP breaks the match on the 7th position, min. match critera is not satisfied.
rs1063320	hsa-miR-148a	6	29798749	C	G	✓	Binds one of the sequences with 8 consecutive matches. SNP breaks the match on the 7th position, min. match critera is not satisfied.
rs1063320	hsa-miR-148b	6	29798749	C	G	✓	Binds one of the sequences with 7 consecutive matches. SNP breaks the match on the 7th position, min. match critera is not satisfied.
rs3134615	hsa-miR-1827	1	40362066	A	C	✓	Binds one of the sequences with 8 consecutive matches. SNP breaks the match on the 6th position, min. match critera is not satisfied.
rs4245739	hsa-miR-191	1	204518842	A	C	✓	Binds one of the sequences with 8 consecutive matches. SNP breaks the match on the 5th position, min. match critera is not satisfied.
rs56109847	hsa-miR-510	3	183824557	A	G	✓	Binds one of the sequences with 8 consecutive matches. SNP breaks the match on 5th position, min. match critera is not satisfied.
rs5186	has-miR-155	3	148459988	A	C	✓	Binds one of the sequences with 7 consecutive matches. SNP breaks the match on 4th position, min. match critera is not satisfied.
rs1434536	hsa-miR-125b	4	96075965	T	C	✓	Binds one of the sequences with 8 consecutive matches. SNP introduces a GU wobble on 8th position (7th of seed), the binding energy is below the cut off.
rs193302862	hsa-miR-24	13	84452863	C	T	✓	Binds one of the sequences with 9 consecutive matches. SNP introduces a GU wobble on 8th position (7th of the seed), the binding energy is lower than cut off.
rs8126	hsa-miR-184	14	103603569	C	T	✓	Binds one of the sequences with 7 consecutive matches. SNP introduces a GU wobble on 6th position (7mer with a GU wobble), the binding energy is below the cut off.
rs12720208	has-miR-433	8	16850399	G	A	✓	Binds one of the sequences with 7 consecutive matches. SNP introduces a GU wobble on 6th position, the binding energy is lower than cut off.
rs13212041	hsa-miR-96	6	78171124	C	T	×	SNP is not in 3’UTR
rs2735383	hsa-miR-629	8	90947269	C	G	×	miRNA is predicted not to bind either of the sequences. There exists a mistmatch on the 4th position regardless of the SNP. SNP further breaks the match on 3rd position.
rs34764978	has-miR-24	5	79924683	A	G	×	miRNA is predicted not to bind either of the sequences. Both have only 6 matches with GU wobble.
rs9341070	has-miR-206	6	152420197	C	T	×	miRNA is predicted not to bind either of the sequences. min. match criteria cannot be satisfied in both
rs67384697	hsa-miR-148a	6	31236683	C	-	×	miRNA is predicted to bind both of the sequences. SNP reduces 11 consecutive match to 9.

mrSNP captures 11 disease-associated SNPs out of 16 SNPs (69%).

In the next set of validation experiments, we tested mrSNP on SNPs obtained from the miRdSNP database [19]. We chose the SNPs that map to the miRNA targets predicted by TargetScan for the miRNAs and genes which are experimentally validated to bind. Note that the effects of these SNPs on binding itself was not specifically evaluated experimentally for all cases. There are 108 SNP-miRNA pairs reported in this database for which the SNP lies in the miRNA target. We filtered out the duplicated pairs and polymorphisms longer than a single nucleotide. After filtering, we obtained 64 SNP-miRNA pairs for study. The results of evaluating the 64 pairs are given in Tables 2, 3, and 4.

**Table 2:** SNP-miRNA pairs reported to disturb miRNA bindings by mrSNP for SNPs - miRNA couples obtained from miRdSNPs

**(a) Pairs captured with the minimum matching criteria**							
**SNP**	**miRNA**	**Chr**	**Position**	**Alleles**		**Success**	**Explanation**
rs8829	hsa-miR-101	7	148504618	C	A	✓	Binds one of the sequences with 8 consecutive matches. SNP breaks the match on 2nd position, min. match critera is not satisfied.
rs28635788	hsa-miR-124	2	47301624	C	T	✓	Binds one of the sequences with 7 consecutive matches. SNP breaks the match on 2nd position, min. match critera is not satisfied.
rs28381252	hsa-miR-224	19	45976504	C	T	✓	Binds one of the sequences with 7 consecutive matches. SNP breaks the match on 2nd position, min. match critera is not satisfied.
rs11782817	hsa-miR-144	8	57074233	A	C	✓	Binds one of the sequences with 10 consecutive matches. SNP breaks the match on 2nd position, min. match critera is not satisfied.
rs11550076	hsa-miR-25	9	110247352	A	G	✓	Binds one of the sequences with 7 consecutive matches. SNP breaks the match on 2nd position, min. match critera is not satisfied.
rs10196117	hsa-miR-124	2	47301624	C	T	✓	Binds one of the sequences with 7 consecutive matches. SNP breaks the match on 2nd position, min. match critera is not satisfied.
rs1143552	hsa-miR-181b	22	33256174	A	G	✓	Binds one of the sequences with 8 consecutive matches. SNP breaks the match on the 3rd position, min. match critera is not satisfied.
rs3733067	hsa-miR-30a	3	52290594	A	G	✓	Binds one of the sequences with 7 consecutive matches. SNP breaks the match on 4th position, min. match critera is not satisfied.
rs35180728	hsa-miR-1	11	118473587	-	T	✓	Binds one of the sequences with 7 consecutive matches. SNP breaks (deletes) the match on 4th position, min. match critera is not satisfied.
rs11557771	hsa-miR-218	14	69341243	A	C	✓	Binds one of the sequences with 8 consecutive matches. SNP breaks the match on 6th position, min. match critera is not satisfied.
rs10055	hsa-miR-30a	16	24835876	T	C	✓	Binds one of the sequences with 8 consecutive matches. SNP breaks the match on 6th position, min. match critera is not satisfied.
rs12635	hsa-miR-197	19	14072442	C	T	✓	Binds one of the sequences with 8 consecutive matches. SNP breaks the match on 6th position, min. match critera is not satisfied.
rs3208684	hsa-let-7g	20	30252805	T	G	✓	Binds one of the sequences with 7 consecutive matches. SNP breaks the match on the 6th position, min. match critera is not satisfied.
rs3208684	hsa-let-7c	20	30252805	T	G	✓	Binds one of the sequences with 7 consecutive matches. SNP breaks the match on the 6th position, min. match critera is not satisfied.
rs5031032	hsa-miR-1	12	102796132	-	T	✓	Binds one of the sequences with 7 consecutive matches. SNP breaks (deletes) the match on 6th position, min. match critera is not satisfied.
rs1063320	hsa-miR-148a	6	29798749	C	G	✓	Binds one of the sequences with 8 consecutive matches. SNP breaks the match on the 7th position, min. match critera is not satisfied.
rs1063320	hsa-miR-148b	6	29798749	C	G	✓	Binds one of the sequences with 7 consecutive matches. SNP breaks the match on the 7th position, min. match critera is not satisfied.
rs1063320	hsa-miR-152	6	29798749	C	G	✓	Binds one of the sequences with 7 consecutive matches. SNP breaks the match on the 7th position, min. match critera is not satisfied.
rs12831	hsa-miR-122	16	30081561	A	C	✓	Binds one of the sequences with 8 consecutive matches. SNP breaks the match on 7th position, min. match critera is not satisfied.
**(b)Pairs captured with the minimum energy threshold**							
**SNP**	**miRNA**	**Chr**	**Position**	**Alleles**		**Success**	**Explanation**
rs9266	hsa-miR-181c	12	25362217	G	A	✓	Binds one of the sequences with 7 consecutive matches. SNP breaks the match on the 8th position (7th of seed), the binding energy (of 6mer) is lower than cut off.
1434536	hsa-miR-125b	4	96075965	T	C	✓	Binds one of the sequences with 8 consecutive matches. SNP breaks the match on the 8th position (7th of seed), the binding energy (of 6mer) is lower than cut off.
rs17026326	hsa-miR-19b	3	30733356	A	T	✓	Binds one of the sequences with 8 consecutive matches. SNP breaks the match on the 8th position (7th of seed), the binding energy (of 6mer) is lower than cut off.
rs17026326	hsa-miR-19a	3	30733356	A	T	✓	Binds one of the sequences with 8 consecutive matches. SNP breaks the match on the 8th position (7th of seed), the binding energy (of 6mer) is lower than cut off.
rs3731562	hsa-let-7d	3	48199877	G	A	✓	Binds one of the sequences with 8 consecutive matches. SNP introduces a GU wobble on 2nd position, the binding energy is lower than cut off.
rs3731562	hsa-let-7g	3	48199877	G	A	✓	Binds one of the sequences with 8 consecutive matches. SNP introduces a GU wobble on 2nd position, the binding energy is lower than cut off.
rs3731562	hsa-let-7c	3	48199877	G	A	✓	Binds one of the sequences with 8 consecutive matches. SNP introduces a GU wobble on 2nd position, the binding energy is lower than cut off.
rs3731562	hsa-let-7b	3	48199877	G	A	✓	Binds one of the sequences with 8 consecutive matches. SNP introduces a GU wobble on 2nd position, the binding energy is lower than cut off.
rs3731562	hsa-let-7a	3	48199877	G	A	✓	Binds one of the sequences with 8 consecutive matches. SNP introduces a GU wobble on 2nd position, the binding energy is lower than cut off.
rs1051780	hsa-miR-34a	17	8063056	C	T	✓	Binds one of the sequences with 7 consecutive matches. SNP introduces a GU wobble on 2nd position, the binding energy is lower than cut off.
rs59564714	hsa-miR-15a	11	73686038	A	G	✓	Binds one of the sequences with 7 consecutive matches. SNP introduces a GU wobble on 3rd position, the binding energy is lower than cut off.
rs16952445/rs1138624	hsa-miR-122	16	30081565	C	T	✓	Binds one of the sequences with 7 consecutive matches. SNP introduces a GU wobble on 3rd position, the binding energy is lower than cut off.
rs1801938	hsa-miR-1	19	2101071	T	G	✓	Binds one of the sequences with 7 consecutive matches. SNP introduces a GU wobble on 3rd position, the binding energy is lower than cut off.
rs6875894	hsa-miR-135b	5	112179965	C	T	✓	Binds one of the sequences with 9 consecutive matches. SNP introduces a GU wobble on 4th position, the binding energy is lower than cut off.
rs6875894	hsa-miR-135a	5	112179965	C	T	✓	Binds one of the sequences with 9 consecutive matches. SNP introduces a GU wobble on 4th position, the binding energy is lower than cut off.
rs73306851	hsa-miR-125b	17	38327577	A	G	✓	Binds one of the sequences with 7 consecutive matches. SNP introduces a GU wobble on 5th position, the binding energy is lower than cut off.
rs11552766	hsa-miR-185	3	49396652	A	G	✓	Binds one of the sequences with 8 consecutive matches. SNP introduces a GU wobble on 5th position, the binding energy is lower than cut off.
rs2664575	hsa-miR-17	20	47862478	G	T	✓	Binds one of the sequences with 7 consecutive matches. SNP breaks the match on the 6th position.
rs10187	hsa-miR-210	12	108962804	C	T	✓	Binds one of the sequences with 7 consecutive matches. SNP introduces a GU wobble on 7th position, the binding energy is lower than cut off.
midrule rs70965446	hsa-miR-141	4	56301355	A	G	✓	Binds one of the sequences with 9 consecutive matches. SNP introduces a GU wobble on 7th position, the binding energy is lower than cut off.
rs8226	hsa-miR-124	22	36677414	G	A	✓	Binds one of the sequences with 7 consecutive matches. SNP introduces a GU wobble on 7th position, the binding energy is lower than cut off.
rs35122558	hsa-miR-155	7	32908099	C	T	✓	Binds one of the sequences with 7 consecutive matches. SNP introduces a GU wobble on 8th position, the binding energy is lower than cut off.
rs55774542	hsa-miR-125b	12	48238167	A	G	✓	Binds one of the sequences with 7 consecutive matches. SNP introduces a GU wobble on 8th position, the binding energy is lower than cut off.
rs59628511	hsa-miR-124	9	140509654	C	G	✓	Binds one of the sequences with 8 consecutive matches. SNP introduces a GU wobble on 8th position, the binding energy is lower than cut off.

**Table 3:** SNP-miRNA pairs predicted not to disturb the miRNA bindings by mrSNP on the SNPs - miRNA couples obtained from miRdSNP because both SNP alleles are predicted to bind the miRNAs

**SNP**	**miRNA**	**Chr**	**Position**	**Alleles**		**Success**	**Explanation**
rs11556953	hsa-miR-133a	1	159888369	A	T	×	Binds one of the sequences with 7 consecutive matches. SNP breaks the match on 2nd position. Binding difference is not reported because miRNA still have another target with 8 consecutive matches near the SNP location.
rs13203	hsa-miR-373	1	145442387	A	C	×	Binds one of the sequences with 7 consecutive matches. SNP breaks the match on 4th position. Binding difference is not reported because miRNA still have another target with 7 consecutive matches near the SNP location.
rs3218074	hsa-miR-424	19	30315176	A	G	×	miRNA is predicted to bind both of the sequences. SNP is on the 1st position, there are 7 consecutive matches in both.
rs36076633	hsa-miR-1	1	159888513	-	G	×	miRNA is predicted to bind both of the sequences. SNP is on the 1st position, there are 7 consecutive matches in both.
rs1059479	hsa-miR-138	1	113243892	A	C	×	miRNA is predicted to bind both of the sequences. SNP is on the 1st position, there are 8 consecutive matches in both.
rs3218074	hsa-miR-15b	19	30315176	A	G	×	miRNA is predicted to bind both of the sequences. SNP introduces a GU wobble on 1st position, there are 8 consecutive matches in both.
rs3218074	hsa-miR-15a	19	30315176	A	G	×	miRNA is predicted to bind both of the sequences. SNP introduces a GU wobble on 1st position, there are 8 consecutive matches in both.
rs3218074	hsa-miR-16	19	30315176	A	G	×	miRNA is predicted to bind both of the sequences. SNP introduces a GU wobble on 1st position, there are 8 consecutive matches in both.

**Table 4:** SNP-miRNA pairs not predicted to disturb the miRNA bindings by mrSNP on the SNPs - miRNA couples obtained from miRdSNP because neither SNP allele is predicted to bind the miRNAs

**SNP**	**miRNA**	**Chr**	**Position**	**Alleles**		**Success**	**Explanation**
rs16952475	hsa-miR-185	15	69018917	C	T	×	miRNA is predicted not to bind neither of the sequences. There are 6 consecutive matches, the binding energy is lower than cut off. The SNP further introduces GU wobble on 5th position.
rs17168525	hsa-let-7b	7	135613262	A	G	×	miRNA is predicted not to bind neither of the sequences. There are 6 consecutive matches, the binding energy is lower than cut off. The SNP further introduces GU wobble on 5th position.
rs1802677	hsa-miR-181b	22	33258866	A	T	×	miRNA is predicted not to bind neither of the sequences. There are 6 consecutive matches, the binding energy is lower than cut off. The SNP further breaks the match on 4th position.
rs56165498	hsa-miR-29a	15	68595075	A	C	×	miRNA is predicted not to bind neither of the sequences. There are 6 consecutive matches, the binding energy is lower than cut off. The SNP further breaks the match on 5th position.
rs57321187	hsa-miR-192	10	79550563	C	T	×	miRNA is predicted not to bind neither of the sequences. There are 6 consecutive matches, the binding energy is lower than cut off. The SNP further breaks the match on 6th position.
rs62062994	hsa-miR-29b	17	48261978	G	T	×	miRNA is predicted not to bind neither of the sequences. There are 6 consecutive matches, the binding energy is lower than cut off. The SNP further breaks the match on 3rd position.
rs62062994	hsa-miR-29c	17	48261978	G	T	×	miRNA is predicted not to bind neither of the sequences. There are 6 consecutive matches, the binding energy is lower than cut off. The SNP further breaks the match on 3rd position.
rs7233791	hsa-miR-124	18	47309884	C	G	×	miRNA is predicted not to bind neither of the sequences. There are 6 consecutive matches, the binding energy is lower than cut off. The SNP further breaks the match on 5th position.
rs73954984	hsa-miR-17	2	111925932	C	T	×	miRNA is predicted not to bind neither of the sequences. There are 6 consecutive matches, the binding energy is lower than cut off. The SNP further breaks the match on 7th position.
rs1804734	hsa-miR-21	1	203278606	A	G	×	miRNA is predicted not to bind neither of the sequences. There are 7 consecutive matches with a GU wobble, the binding energy is lower than cut off. The SNP further breaks the match on 6th position.
rs3802782	hsa-let-7b	11	69468919	T	C	×	miRNA is predicted not to bind neither of the sequences. There are 7 consecutive matches with a GU wobble, the binding energy is lower than cut off in both. The SNP is on 11th position, it further reduces the binding energy.
rs3218074	hsa-miR-503	19	30315176	A	G	×	miRNA is predicted not to bind neither of the sequences. There are 7 consecutive matches with a GU wobble, the binding energy is lower than cut off. The SNP further introduces GU wobble on 1st position.
rs1803045	hsa-miR-1	12	49330252	C	T	×	miRNA is predicted not to bind neither of the sequences. There are 6 consecutive matches with a GU wobble, min. match criteria cannot be satisfied in both. The SNP is on 12th position, it further reduces the binding energy.

As Table 2 shows, mrSNP reports the binding effects of 43 SNPs out of 64 (67%) couples. For these miRNA-mRNA couples, the SNPs either disrupt a match in the seed region or introduce a new GU wobble. For 19 of these pairs given in Table 2(a), the SNPs break a matching in the seed region, therefore, the minimum matching criteria cannot be satisfied. On the other hand, 4 of SNPs given at the top of Table 2(b) break a matching at the end of the seed region, resulting a 6mer, for which the binding energies become lower than the threshold. Similarly, the other 20 SNPs in Table 2(b) introduce GU wobbles in the seed region, resulting to disturb the binding due to the minimum binding energy criteria.

Table 3 lists the 8 pairs for which mrSNP does not report a binding difference as the sequences for both alleles are predicted to bind the miRNAs at similar levels. Note that for the first two pairs in the Table 3, mrSNP captures the disruption in binding. However, mrSNP does not report these SNPs to affect binding, as it identifies another seed region for the miRNA in a location very close the original target. mrSNP identifies changes in the binding energies of the pairs in Table 3, which are ignored as the sequences for both alleles satisfy the minimum matching criteria. Table 4 lists another category of pairs that were not predicted by mrSNP. For these 13 pairs, the sequences for neither allele were calculated to bind the given miRNAs. The binding of these miRNAs are not predicted because the minimum matching criteria is not satisfied, as explained in more detail in the table.

We queried the 64 SNPs (Tables 2, 3, and 4) on mirSNP, PolymiRTS, and mirsnpscore. MirSNP reports 44 of these pairs as binding difference, and the results of MirSNP are very consistent with mrSNP. 57 of the 64 pairs queried are present in the PolymiRTS database, which includes miRNA-mRNA pairs identified through methods that include experimental data such as gene expression profiles and cross-linked immunoprecipitation sequencing data as well as pairs identified from the literature (rather than purely prediction methods). Because of the inclusion of experimental data and results from the literature, it is difficult to compare the results of PolymiRTS to mrSNP. Only 9 of the validated miRNA-mRNA pairs are found using mirsnpscore. Note that although the miRNAs-gene bindings are experimentally validated in this experiment, (Tables 2, 3, and 4), the actual effects of the SNPs on miRNA bindings are unknown. Therefore, it is not possible to determine the biological accuracy of the tools in this experiment. However, one result we can conclude from this experiment is that, mrSNP captures 51 (43 + 8) of the 64 (80%) experimentally validated miRNA bindings.

When comparing mirSNP to mrSNP across both experiments, 56 of 80 SNPs (70%) were predicted by mirSNP to disrupt miRNA binding. mrSNP compares favorably by predicting that SNPs will disrupt the binding 54 of the 80 (68%) miRNA target sites across these two experiments.

## Conclusion

We developed a new tool, mrSNP, that predicts the effects of SNPs on miRNA binding. There are several advantages to this tool over existing tools. The proposed tool not only works on existing SNPs in databases such as dbSNP but also on novel SNPs which will be of great utility for researchers identifying new SNPs or somatic mutations in their samples. Secondly, our tool decreases the manual labor currently required for running prediction algorithms for novel SNPs. We present the results of mrSNP for various 3’UTR SNPs that were experimentally validated to disturb miRNA binding. We also compare the performance of mrSNP with other miRNA binding prediction tools, for which mrSNP performed better than all but one other platform, MirSNP, that had a success rate of 75% (Table 1). mrSNP correctly predicted 11 of 16 (69%) disease-associated and/or experimentally validated SNPs that are reported in the literature or other databases. We observed that the recovery rate of mrSNP can be adjusted by using different set of parameters, but this may alter the false-positive rate. The major limitation of mrSNP is that it did not capture all of the SNPs experimentally predicted to disrupt miRNA binding. In future experiments, we will study additional larger sets of experimentally validated SNPs to improve the sensitivity and specificity of our binding predictions. As the literature is beginning to note miRNA binding to other regions of mRNAs and the potential for an influence on the 3’UTR location on binding, we will strive to incorporate these into our algorithms. In summary, mrSNP is a highly adaptable and performing tool for predicting the effect a SNP will have on miRNA binding.

## Availability and requirements

**Project name:** mrSNP;

**Project home page:**http://mrsnp.osu.edu;

**Operating system(s):** Platform independent;

**Programming language:** Python, PHP and JavaScript;

**Other requirements:** JavaScript compatible browser;

**License:** Free for commercial and academic use;

**Any restrictions to use by non-academics:** No specific restrictions.

## Competing interests

The authors declare that they have no competing interests.

## Authors’ contributions

AET and UVC conceived of the study, and participated in its design and coordination and helped to draft the manuscript. MD developed the workflow, ran the experiments, and wrote the manuscript. All authors read and approved the final manuscript.
